# Caring and Uncaring Encounters between Assistant Nurses and Immigrants with Dementia Symptoms in Two Group Homes in Sweden-an Observational Study

**DOI:** 10.1007/s10823-018-9351-y

**Published:** 2018-06-21

**Authors:** Mirkka Söderman, Sirpa Rosendahl, Christina Sällström

**Affiliations:** 1 School of Health, Care and Social Welfare, Mälardalen University, Box 325, SE-63105 Eskilstuna, Sweden; 2 Division of Insurance Medicine, Department of Clinical Neuroscience, Karolinska Institutet, SE-171 77 Stockholm, Sweden; 3 School of Health and Education, University of Skövde, Box 408, SE-541 28 Skövde, Sweden; 4 Faculty of Health, Science and Technology, Department of Health Sciences, Karlstad University, 651 88 Karlstad, Sweden

**Keywords:** Caring, Dementia, Encounter, Immigrant, Qualitative, Uncaring

## Abstract

Background: The total number of people with dementia symptoms is expected to double every 20 years and there will also be an increase in the number of older immigrants in several countries. There are considerable deficiencies in the present knowledge of how to conduct well-functioning health care for immigrants with dementia symptoms. The aim of this study was to explore caring and uncaring encounters between assistant nurses and immigrants in two group homes for persons with dementia symptoms in Sweden: a Finnish-speaking as well as a Swedish-speaking context. In addition, this study aims to describe how caring and uncaring encounters are manifested in *these* two contexts according to Halldórsdóttir’s theory of “Caring and Uncaring encounters”. Method: Descriptive field notes from 30 separate observations were analyzed using qualitative deductive content analysis. Results: The main category “caring encounters” focused on reaching out to initiate connection through communication, removing masks of anonymity by acknowledging the unique person, acknowledgment of connection by being personal. Reaching a level of truthfulness by being present and showing respect, raising the level of solidarity by equality and true negotiation of care, based on the residents’ needs. The main category, uncaring encounters, focused on disinterest in and insensitivity towards the other, coldness in the connection and lack of humanity in care situations. The observations showed that caring encounters occurred more in the Finnish-speaking context and uncaring encounters more often in the Swedish context**.** Conclusion: Encounters could be caring, uncaring, and carried out using a person-centered approach. Communication and relationships could be facilitated using the same language but also through learning to interpret residents’ needs and desires.

## Introduction

Globally, there are an estimated 35.6 million people living with dementia, and this number is expected to double with every 20 years (Prince et al. 2013). Several English-speaking countries also have an ageing immigrant population and increasing groups with non-English speaking older people living with dementia, such as in Australia (Boughtwood et al. 2011; Runci et al. 2005), the USA (Kong et al. 2010) and the UK (Lawrence et al. 2011). In Sweden, with a total of 10.1 million inhabitants (Central Bureau of Statistics 2018), 20,000 of 160,000 people living with a dementia diagnosis have foreign backgrounds. There might also be a large number of unreported cases since cultural aspects can become barriers to seeking health care for dementia symptoms (Haralambous et al. 2014; Kong et al. 2010). Even if the influence of cognition and language affects all people with dementia symptoms, the behaviors, reactions and responses may be influenced by the patient’s native culture. Having knowledge about a person’s language, cultural traditions and early life could be a way to understand the immigrant with dementia (Hanssen 2013). This can become a challenge for healthcare professionals in their daily work activities.

## Communication in the Care of Older Immigrants with Dementia Symptoms

Dementia is a symptom complex that, according to the DSM-5, is classified as either a mild neurocognitive disorder (NCD), with milder disabilities, or a severe NCD, with greater care needs (American Psychiatric Association 2013). Irrespective of subtype, the behavioral and psychological symptoms of dementia are a large part of the dementia syndrome and have a great relevance because their occurrence follows the degree of both functional and cognitive impairments. For patients, the symptoms have an impact on their quality of life (Cerejeira et al. 2012).

Language impairments are common and can affect both the linguistic ability and/or understanding of language for immigrants with dementia symptoms (Tang-Wai and Graham 2008). The meaning of multilingualism in later life varies; older people might need to use their mother tongue despite knowledge of foreign languages, whereas others alternate between languages or continue speaking the foreign language. For an adult who learns a second language in the context of migration, this concerns being part of a sociolinguistic landscape with limitations of varying degrees (Divita 2014). Health care can be seen as such a sociolinguistic landscape in which older immigrants with dementia symptoms often are cared for in units where none or only a limited number of caregivers speak the same language as the older patients (Runci et al. 2005; Wu et al. 2010). This can lead to isolation and feelings of loneliness (Mazaheri 2013; Runci et al. 2005). A previous study of Finns with dementia symptoms showed difficulties in communicating with the Swedish-speaking assistant nurses (AN), even when communication was adequate in their mother tongue with bilingual AN (Ekman 1994; Söderman and Rosendahl 2016). In addition, when patients shared language with AN, they had a higher level of competence, their integrity was preserved better and their quality of life increased when they were cared for by bilingual AN (Ekman 1994; Söderman and Rosendahl 2016). In a study of multilingual encounters, the limitations of communication in care activities for a Persian woman with dementia symptoms lead to a risk of incorrectly interpreting her symptoms and needs (Plejert et al. 2014), which has been seen also in a study on Finns (Söderman and Rosendahl 2016). Being sensitive to and understanding patients’ nonverbal expressions is important in achieving a high quality of care (Sellevold et al. 2013). Both verbal and non-verbal behavior can be used as a reaction to or to avoid undesirable situations related to language limitations, which makes it important to provide education in communication for caregivers (Small et al. 2015). In our multicultural society caregivers need to be creative in multilingual encounters by, for example, using some learned phrases or using a common understanding of languages that are so alike that it is possible to understand each other. Language matching is an option when the availability of a shared language is limited (Jansson 2014).

## Encounters in Daily Caring

In Halldórsdóttir’s (Halldórsdóttir 1996, 2008) theory, a caring professional is characterized by competence, caring and connection. Caring encounters can be symbolized by a bridge built by the professional; the bridge symbolizing closeness and distance between a nurse and patient, openness in communication and connection in the care experienced by the receiver, which affects the receiver in a positive way, ultimately leading to empowerment. This in turn results in an increased sense of quality of life for the patient. When caring is marked by incompetence, indifference and distrust, there is no connection. Uncaring encounters is symbolized by a wall; when there is no communication and connection, there is separation instead, which affects the receiver in a negative way because this fuels discouragement. Caring becomes uncaring and lowers the sense of quality of life for the patient. It is also emphasized that this is not about a dichotomy, but different ways of relating to each other (Halldórsdóttir 1996, 2008). In caring, it is important to see the patient both in an inner and outer context. The inner context deals with the perceived needs, expectations of the patient and also the patient’s experience and self-esteem. This can be summarized as the feeling of vulnerability and need for professional care. The patient’s outer context also includes the perceived surroundings, which merges with the nurses’ context (Halldórsdóttir 1996). Furthermore, Halldórsdóttir (Halldórsdóttir 1996, 2008) describes different phases of caring encounters required to build the bridge. These encounters involve reaching out and initiating connection, removing the mask of anonymity and acknowledgments of connection. These encounters also involve reaching a level of trustfulness and solidarity, and finally, true negotiation of care. The wall that rises between the nurse and the patient in uncaring encounters is built upon a lack of interest in and insensitivity to the other person, coldness in the connection and a lack of humanity. Uncaring encounters can occur as a result of many factors and potentially hold back nurses’ ability to connect and understand both themselves and their patients. It is therefore important to recognize this dark side of nursing (Adams and Maykut 2015).

Through a caring relationship the patient and the nurse create an encounter in the process of daily care activities. In addition, by reciprocity, there is an overall vision of care that reduces the patient’s sense of vulnerability and maintains dignity (Berg 2006). The relationship based on this reciprocity is the basic structure of care in which the patient is seen as a unique individual, and during the encounter, a connection between the patient and the caregiver is formed (Fredriksson 2003). A care culture in which the development of relationships and respect for the caretaker’s subjective life-world is secondary, could lead to alienation and uncaring, and this can also happen when routines do not consider a patient’s uniqueness (Rytterström 2011; Wiman and Wikblad 2004).

When care is based on a person-centered approach, it is based on communication and relationships, which provide the opportunity for nurses to have knowledge of a specific individual’s life-history and abilities in daily life. Through this, nurses can support people with dementia symptoms in maintaining independence and can maintain this for longer periods of time than in other forms of care (Sjögren et al. 2013). The overall objective in the care of people with dementia symptoms is to protect and maintain their quality of life (Sellevold et al. 2013; Sjögren et al. 2013).

In the very near future, many countries will host more multilingual older people who are in need of health care interventions, and the issue of dementia in non-native language speaking persons cannot be postponed (Plejert et al. 2014; Söderman and Rosendahl 2016). Furthermore, it is important to examine if older people with different ethnic backgrounds have specific needs, to offer culturally congruent care (Kong et al. 2010; Runci et al. 2005). Although nurses supplement their verbal communication with non-verbal communication, the question remains how to provide these older individuals with well-functioning health care. Knowledge concerning the influence of ethnicity and language on care encounters remains limited. Further research in this area could provide a deeper understanding of the care requirements for encounters based on cultural differences to enhance the quality of life for residents living in group homes.

## Aim

The aim of this study was to explore caring and uncaring encounters between AN and immigrants in two group homes for persons with dementia symptoms in Sweden: a Finnish-speaking and a Swedish-speaking context. In addition, this study aims to describe how caring and uncaring encounters are made manifest in *these* two contexts according to Halldórsdóttir’s theory of “Caring and Uncaring encounters”.

## Material and Method

### Design

This study is a part of a larger study entitled “*Ethnic minority people with dementia being cared for in special accommodation in Sweden*”. The data was collected during a period of one year which also included interviews with relatives and AN (Rosendahl et al. 2016). An observational study with a qualitative approach and a qualitative, deductive content analysis method was chosen. Observation as a data collection method is useful because researchers can observe participants’ behavior and actions that might not be reported in self-reports (Polit and Beck 2011). The researcher is, during observations, involved in a variety of activities over a long period which makes it possible to follow daily life. The process of observations facilitates the understanding of behaviors and activities and the development of a story that explains various aspects (Kawulich 2005). The analysis will follow Elo and Kyngäs’ (Elo and Kyngäs 2008) description of qualitative deductive content analysis. In the analysis process Halldórsdóttirs’ (Halldórsdóttir 1996, 2008) theory of caring and uncaring encounters in a new context, is applied to the care of persons with dementia symptoms in order to develop a structured analysis matrix according to the concepts of the theory. The analysis also determines how the concepts of the theory are manifested in two linguistically diverse contexts.

### Study Context

In Sweden people with severe dementia symptoms are often cared for in group homes that consists of smaller units with higher workforce ratio than ordinary nursing homes. The group homes in which the observations were carried out were designed for 10–11 care-takers with dementia symptoms, according to the Swedish regulations for being granted placement in this type of accommodation (The National Board of Health and Welfare 2010). Not everybody in this study had a dementia diagnosis, but all of them showed symptoms of middle to advanced stage of dementia. Therefore, no scale has been used to measure the dementia symptoms. In the Swedish context, the participants seemed to have reached more advanced stages due to the level of support observed during the daily activities. The nursing staff in each group home were AN, and one registered nurse had the responsibility for several wards. All AN had vocational education in nursing care including three years of secondary education and had equivalent duties. Each resident had a designated AN who functioned as a contact person with a main responsibility for that specific resident. Group homes consisted of common areas, such as a day room and a dining room, and individual rooms with en-suite bathrooms. Care in group homes was not expressly person-centered but, as the observer could notice, shaped such as to keep the individual requirements in focus. Observations were carried out in all areas of the group homes.

### Participants

All persons who lived, worked or visited (relatives) places where the observations were carried out, were asked to participate in this study. In the Swedish-speaking group home, only non-Swedish speaking residents as well as staff members were asked to participate, as shown in Table 1. All those who were asked to participate, agreed to participate and were included in the study. The residents who were immigrants consisted of 4 men and 13 women, with the age-range from 70 to 90 years and were of Slavic origin. They had immigrated to Sweden as workers or refugees after The Second World War and all except two, had spoken Swedish fluently before the dementia disease affected their skills in the second language. All 11 residents in the Finnish-speaking group home were observed (Table 1) while in the Swedish-speaking group home only the non-Swedish speaking residents were observed. The staff in the Swedish-speaking group home comprised 28 AN, 2 men and 26 women, with the age-range from 20 to 60 years of Swedish, Finnish, Estonian, Hungarian and Ethiopian origin. The staff was Swedish-speaking, with Swedish either as their mother tongue (Table 1) or as a second language.

**Table 1:** Participants

Context		Finnish speaking group home in Sweden	Finnish speaking group home in Sweden	Swedish speaking group home in Sweden	Swedish speaking group home in Sweden
Characteristics		Nurses	Resident	Nurses	Resident
Language	Mother tongue other than Swedish	12	11	5	5
	Swedish	0		10	–
Age	Range	20–58	70–90	23–60	70–90
Gender	Male	2	4	0	0
	Female	10	7	16	6
Total number		12 nurses	11 residents	16 nurses	6 residents

### Data Collection

After permission was granted from both the Ethics Review Board and operation managers, contact was initiated by the second author (SPR) with head nurses at their respective work places. The head nurses then introduced the second author (SPR) and the study to the participants. Participants, AN and residents, were given verbal and written information about the study and its purpose and were informed that participation was voluntary and that they could withdraw from the study at any time. For all immigrant residents with dementia symptoms, informed consent was requested from relatives. The overall purpose of the first observation was to get an overview of the context, the environment and the interior of the group home, the people and objects. Descriptive field notes were collected both during and immediately after the observation (cf. Polit and Beck 2011). In addition, questions and inquiries, which arose during the observation, were noted and followed up after each observation with the AN. The second author (SPR) who is also trilingual - speaking Swedish, Finnish and English - was at the group homes for up to 2 h at a time, and each individual observation lasted from 5 min - 30 min during different days and times of the day. Observations were carried out in residents’ rooms and bathrooms, during mealtime in the dining room and celebrations in the dayroom. The focus of the observations was communication and interaction between the residents and AN. The intention was to observe different residents and AN, but the variation in AN depended on who was on duty. During breakfast and dinner observations, the observer (SPR) sat on a chair at the side and watched the residents and AN. During activities of personal hygiene, the observer (SPR) chose to stay outside of the restroom out of respect for the residents; the observer (SPR) heard the conversation but did not see the interaction. Informal conversations with the AN was also performed to confirm that observations were correctly interpreted. In connection with the observations, a diary was kept in order to maintain the role as researcher and maintain distance from the persons observed. The data consist of a total of 30 different observation occasions. In the Finnish-speaking group home, the observations resulted in a total of 228 notes, and in the Swedish-speaking group home, there were a total of 181 notes.

### Data Analysis

According to Elo and Kyngäs’ (Elo and Kyngäs 2008) description of a deductive content analysis (cf. Andersson et al. 2015), and after the preparation phase and selecting the unit of analysis, step one consisted of making sense of the data in its context. This was performed when field notes from the observations were read through several times for a better overall understanding and to allow immersion in the material. Step two, developing a structured analysis matrix, consisting of an operationalization of phases in Halldórsdóttirs’ (Halldórsdóttir 2008; Halldórsdóttir 1996) theory of caring and uncaring encounters, was performed, as shown in Table 2. To obtain the core of caring and uncaring encounters, a structured categorization matrix was created from phases and aspects that were established and validated through discussion between the first (MS) and third (CS) author, as shown in the example in Table 3. Every aspect of the different phases was numbered to facilitate further analysis.

**Table 2:** Phases in caring and uncaring encounters

Caring encounters	Uncaring encounters
Reaching out: initiating connection	Disinterest
Removing the masks of anonymity	Insensitivity
Acknowledgment of connection	
Reaching a level of truthfulness	Coldness
Reaching a level of solidarity	
True negotiating of care	Inhumanity

**Table 3:** Analysis matrix

Main concept	Phases in caring encounters	Aspects of phases
Caring encounters	Reaching out: initiating connection	1.Taking contact through conversation
		2. Touching the other
		3. Seeking for eye contact with the other

The next, step 3, data coding according to the categories was performed, by selecting codes that were consistent with the categorization matrix (cf. Elo and Kyngäs 2008). The codes were transfered to a code sheet; the text was converted to a table and the codes were sorted by each aspect to various headings. Step 4, consisted of hypothesis testing, correspondence comparison to earlier studies by using an iterative process between the text and the codes as seen in the categorization matrix. During step 5, reporting the analyzing process and the results by a model, the main categories were named according to Halldórsdóttir’s theory’s (Halldórsdóttir 1996, 2008) two aspects of caring: caring and uncaring. Then, the categories were named after the phases in the theory, and the subcategories were named after groupings based on similarities in aspects, as shown in Table 4. All steps in the analysis were discussed between the first (MS) and the third (CS) author until consensus was reached. The field notes collected by one team member (SRP) were reviewed and discussed by all authors.

**Table 4:** Analysis procedure

Main category	Category	Subcategory	Code
Caring encounters	Reaching out: initiating connection	To touch the other	Nurse asks residents what this done with food and hugs while doing it
			Nurse hugs resident for good night

## Ethical Considerations

The study was approved by the Regional Ethical Review Board at Linköping University Sweden (Dnr 03–264), (Dnr 02–053). During the observations, it was the researcher’s intent to be responsive if the residents became concerned about the researcher’s presence. This was done by observing changes in the residents’ moods and being responsive to the residents’ wishes. The data have been stored to ensure that unauthorized persons do not have access to it, and all names and places are fictitious to protect participants from being identified.

## Results

The analysis revealed that the main category of *caring encounters* involved encounters portrayed during the observations as reaching out when one of the parties initiated a connection, removing the mask of anonymity and recognizing contact. Furthermore, these encounters were portrayed as reaching a level of sincerity and solidarity and true negotiation of care. The main category of *uncaring encounters* involved encounters portrayed as showing a lack of interest in or indifference to the other person; these encounters were seen as having insensitivity or coldness and also a lack of humanity. Every category in the result section is preceded by a description from the theory before describing the observation or result of analysis.

### Caring Encounters

In the main category of “caring encounters”, observations led to a description of how these encounters were shaped. Caring encounters were formed by reaching out when one of the parties initiated connection, by removing masks of anonymity and by acknowledging contact. Furthermore, these encounters could be achieved by reaching a level of truthfulness or solidarity and by true negotiation of care. A caring encounter is described by the following encounter between an AN and a resident: The AN asks the resident in shared language, “Should we shave, where is it (the shaver)?”, and then turns on the shaver and gives it to the resident, who does the shaving himself. The resident shaves hesitantly and slowly and asks, “Is the beard coming off?” The AN replies, “Yes, but you have some left under the chin” and sits on the bedside. She tells him where he should shave and says, “Now I think it’s fine” and receives the shaver from him to clean it. There is a connection, a togetherness, between the AN and the resident, and the AN helped the resident according to the resident’s need.

#### Reaching Out - Initiating Connection

The first category, “reaching out - initiating connection”, means creating an opportunity for a connection through verbal and non-verbal communication. In the analysis, this emerged through contact involving conversation, touching the other person or searching for eye contact with the other person.

During mealtime observations, it was noted that AN initiated conversation by asking questions to the residents when serving food or noticing if any of the residents had a poor appetite. Similarly, conversations were initiated during personal care; for example, an AN approached a resident and asked, “Can we help you? We thought we’d help you go to bed.” Bilingual AN also made connections by greeting residents using their mother tongue.

The connection was also initiated by touch, as the AN gave residents a hug at mealtime or bedtime, or when they greeted residents. Observations revealed that other ways of initiating a connection was by the AN seeking eye contact when they asked residents questions and by using body language. Observations revealed that even the residents used body language to clarify their verbal communication. A connection was also noted when the resident sought contact with the contact person when she was not in sight during mealtime.

#### Removing the Masks of Anonymity

The category “removing the masks of anonymity” involves removing stereotypes for a mutual acknowledgment of each other as persons and unique individuals, and accepting each other; in the analysis, this emerged through the other person being called by name. The observations showed that the AN called the other person, the resident, by name during meals and when caring for and greeting residents. This was done in a respectful manner when the AN addressed residents by both using the first name and addressing them as “Auntie”, which in the Finnish context, is used to show respect for someone older: “Here we have for Auntie-(resident’s name)…”.

#### Acknowledgments of Connection

The next category, “acknowledgement of connection”, means that the AN responds personally and makes eye contact but also uses body language and has a warm tone of voice. The analysis revealed that AN and residents used humor during contact with each other, that they related to themselves and confirmed the other person. Acknowledgements of connection also emerged through being gentle with touching and using a warm tone of voice when speaking to residents.

During the observations, AN were personal in conversations with the residents by making humorous comments at mealtime and during personal care. Residents in turn responded to the AN’s comments with laughter and their own jokes. This could also occur in situations when the AN followed a resident, who walked with a walker to bed and both fell on the bed, which made both the AN and resident burst into laughter. At mealtime, the resident could reply to the AN with a laugh and the AN would speak jokingly; for example, when one resident dropped her sandwich in her coffee, the AN said, “Now we need to do some fishing here.”

The observations also revealed that residents were personal in their conversations with AN, by relating to themselves when they expressed their wishes and needs or commenting on their bodily activities; for example, one resident who happened to burp said, “That means I should stop eating now. You should not eat too much, it’s not healthy.” It also emerged that during mealtime, residents told the AN about their childhood or their previous professional life and during conversations, a resident would tell the AN that she had a doctor’s appointment for her bad hip.

During the observations, acknowledgement of connection could occur when residents confirmed an AN when responding to the AN’s questions at meal times and when receiving personal care. Confirmation of the other was also seen when a resident who ate with her fingers began to eat with a spoon instead, after heeding an AN’s suggestion. Another example is when an AN sat at the table and took apart a Russian nesting doll that was placed as an ornament on the table and the resident followed this action with interest.

The observations revealed that acknowledgement of connection could occur when the AN showed gentleness in touch during their movements and when helping residents into bed for the night. This was also seen when the health of residents changed, for example, when an AN suddenly stood up and walked over to a resident who did not eat to feel his forehead: “He feels hot. Maybe he has a fever?” A gentle touch was also observed when the contact person and residents strolled hand-in-hand or when the AN placed a resident’s hand on a glass so she could drink by herself. Acknowledgements of connection was also observed when the AN spoke to residents respectfully with warmth in their voice during conversations and encouragement as well as when they wished them good night at bedtime.

#### Reaching a Level of Truthfulness

The category “reaching a level of truthfulness” concerns the ANs’ willingness to be present even in troublesome situations and the ANs’ respect for the resident, which in the analysis was seen by the AN being present during meals and having a common understanding with residents. Furthermore, observations showed that the AN confirmed the residents’ feelings but also that residents showed honesty with the AN.

In the observations, willingness to be present was shown by an AN sitting with residents at the table and eating together at meals. In observations, AN and residents sharing a similar world-view could be seen by residents being compliant with AN assistance at mealtime. During personal care, this was shown by an AN who perceived a resident’s resistance to the removal of a blanket as she felt cold, and saying, “Now we have to remove the covers…Is it cold?” AN and residents, under observation, conversed about food or events in the residents’ everyday life, such as having received a pedicure. However, the conversations also revolved around events far back in time involving the residents’ childhood. Resident: “I had a wonderful childhood.” AN: “Do you have any siblings?” Resident: “I have siblings, five sisters and brothers.” Furthermore, residents could express their desire for food to a AN, and the AN could understand. Even though the residents had reduced language skills and incoherent speech; the resident could still manage to express that he/she wanted a sandwich. By speaking reassuringly to residents when they were sad, the AN confirmed the residents’ feelings. Residents, in turn, could demonstrate honesty towards the other person when realizing that they had given inadequate answers and telling the AN about this; for example, one resident who had given the names of her siblings corrected herself when she realized those were the names of her own children.

#### Reaching a Level of Solidarity

The category “reaching a level of solidarity” means that the feeling of experiencing alienation disappears and is replaced by the experience of equality. In the analysis, this emerged by the demonstrated concern for the other person, the interpretation of that person’s needs and the importance of a shared language.

During the observations, the AN gave instructions and repeated suggestions until the residents gave feedback. The AN also guided residents to do things the right way during the meal and helped the residents using advice and assistance, for example with food intake when a resident asked for help cutting food into smaller pieces. The AN supervised residents’ food intake and were attentive of residents who needed napkins to wipe their hands when they had eaten with their fingers. The ANs were also concerned about changes in the residents’ health and facilitating a good night’s sleep for residents; this was shown by the AN helping to remove jewelry that could press against a resident’s cheek during the night with a hug or by wishing residents a good night, “Good night, sweet dreams,” resulting in the resident calmly laying down in bed.

Solidarity was also created using a shared language. Bilingual ANs talked to the residents in the mother tongue even when a response was left out or when the responses to the mother tongue were inadequate because of the disease. The AN’s greetings in the mother tongue or in closely related languages were answered verbally or with a smile by the residents.

#### True Negotiating of Care

The last category in caring encounters, “true negotiating of care”, involves the AN working with the resident and working to be supportive, which in the analysis was shown by AN who helped residents based on the residents’ individual needs, and showed respect for the residents and maintained the residents’ dignity.

During the observations, AN understood residents’ needs, which was seen by AN speaking slowly and clearly so residents could understand. The AN could verbally coax and try to distract resisting residents, but this did not always succeed; for example, an AN tried to help one resident with her hygiene, and she resisted by squeezing her legs together. The AN gave instructions and support based on residents’ needs during mealtime and personal care. For example, an AN supported a resident’s self-care by giving instructions for shaving that the resident managed to follow.

The observations also revealed that as a way of showing respect, AN told the residents what would happen and how; for example, an AN said slowly, “Now you’ll have to stand up again, then we will put you in bed.” The process ran smoothly, and the resident got into bed. Respect was also shown when residents were addressed by name during both food service, making one’s toilet and when care was cancelled out of respect for residents who were being resistant; for example, one resident did not want dentures to be put in her mouth, so the AN stopped and said, “Okay, then we’ll do it later”. During observations of the care of residents who seemed not to understand the Swedish language, the AN at all times explained in Swedish what was being done, and the resident could either be compliant or resist the AN. At mealtimes, the AN could even excuse their lack of attentiveness to the residents’ wishes for support by carrying away their plate: Resident: “No, no, can you take it or not?” “Was I supposed to take it? I didn’t hear,” the AN says, and she excuses herself. The analysis also showed that the AN were able to maintain the residents’ dignity by guiding them in a humble way; for example, as one resident began to fuss with the tablecloth, the AN said, “You know what, (resident’s name), we’ll let the cloth lay on the table right now,” and the resident understood this way of requesting. The AN also maintained the residents’ dignity by ensuring that residents had a nice appearance; for example, an AN went over to a resident who had spilled on her blouse and wiped the spill before she left the table.

#### Differences Between Contexts in Caring Encounters

The analysis revealed that *caring encounters* occurred in both contexts (95% of notes in the Finnish context, 77,9% of notes in the Swedish context). There were also differences between the contexts in that humor was used hardly at all (1,1% of notes) in the Swedish-speaking context but on several (10,9% of notes) occasions in the Finnish-speaking context. AN and residents shared a life perspective in both contexts, but it appeared more frequently in the Finnish-speaking context (8,3% of notes) compared to the Swedish-speaking (5,5% of notes) context. The importance of the shared language, as a way of reaching togetherness, was only observed in the Swedish-speaking context (7,1% of notes). In the Finnish-speaking group home, residents and AN spoke the same language. Both consideration for the other person (7,8% of notes in the Finnish context, 3,3% of notes in the Swedish context) and the desire to help based on the other person occurred in both contexts, but differed in the number of occasions (17,5% of notes in the Finnish context, 12,1% of notes in the Swedish context). Respect for the other person also occurred in both contexts but differed in number (3,9% of notes in the Finnish context, 9,3% of notes in the Swedish context).

### Uncaring Encounters

In the main category of “uncaring encounters”, observations led to a description of how these encounters were shaped. Uncaring encounters occurred when a person showed a disinterest or indifference to the other person, and these encounters were characterized by insensitivity or coldness and a lack of humanity. The observations revealed that uncaring encounters could occur at mealtimes when there was an absence of socializing at the table where residents sat. Residents sat quietly and ate, they were served food and had help with medication, but the AN talked to the residents only when necessary; for example, when serving food, the AN would ask if the residents wanted more, but there was no further conversation. AN showed a lack of interest in and a coldness toward the residents when their tasks were performed routinely.

#### Disinterest in the other

The category “disinterest in the other” means that the AN has a limited attentiveness to the resident’s specific needs, has limited communication with the resident and shows an unwillingness to have contact. According to the background theory, this reflects a lack of caring more than the existence of uncaring. In the analysis, this was shown by residents not being invited into conversations or not being addressed by the AN and was also seen by AN holding conversations that residents seemed not to understand.

During observations, not being invited into the conversation occurred when an AN was speaking so rapidly that residents with insufficient language ability had difficulty understanding what was said. In observations, a lack of interest for the other person was also seen during meals when residents were not asked about drinks (the AN instead served everyone the same drink) or when meals were eaten and personal care was delivered in silence. The AN would talk to each other during the meal, and even during the observation, conversations would occur that excluded the residents. AN spoke with the residents as much as with each other, but a resident who ate in silence became angry by the fact that AN’s were talking to each other.

#### Insensitivity for the other

The second category, “insensitivity for the other”, involves a difference between the AN and the person receiving care. Insensitivity means that the AN seemed not to notice the resident’s feelings and is perceived as heavy-handed by the resident, and that the resident has the experience of disturbing the AN. In the analysis, this was revealed by a lack of the AN’s presence, a lack of response from the residents or AN, a limited amount of information given to the residents and hard-handedness in the AN’s touch.

During the observations, the lack of presence was shown by the AN being busy serving during meals and by AN who took their breakfast at another table rather than sitting with the residents. During the observation, the information from the AN was restricted because they asked questions to residents and told them what should be done, but the residents did not understand due to limitations in the language ability.

During the observations, the residents did not respond to the ANs’ questions during the meal or when receiving personal care. In turn, an AN might ask a question to a resident with limited language ability: AN: “Do you want a sandwich?” The resident replies “No,” and looks confused. Even when spoken to in the mother tongue the response could be withheld by the resident; for example, an AN asked about a visit from relatives the day before: “Was your son here yesterday on your birthday? Do you remember what happened yesterday?” When the resident did not respond to the AN question, the AN continued,“ No, you probably won’t remember it”. During one observation, when an AN was going to remove a resident’s dentures for the night and she did not want to open her mouth, that AN had to use mild force to remove the teeth from her mouth.

#### Coldness in Connection

The category “coldness in connection” means that contact is business-like and technical from the AN, and the resident feels as though the AN would be better off without the resident. The analysis of routine work revealed this.

The observations showed that during routine work, the AN would serve food portions without asking residents about the size of the portion, and the AN would speak quickly with residents, which resulted in them resisting; for example, while delivering care, an AN spoke quickly and said to a resident, “You should wash your face, my mother tells me that,” and the resident responded with shouts of “No, no.” This was also seen when an AN gave a resident her medicine mixed in the food, and although she grimaced badly when ingesting it, there were no further comments.

#### Lack of Humanity

The last category, “lack of humanity”, involves the AN ignoring the resident, shortcomings in the care of the resident or ridiculing the resident. With lack of humanity there is an increased risk that residents experience that the AN is against them and has no respect for them. The analysis revealed that the residents were treated against their will and with a lack of respect.

In the observations, being cared for against one’s will was seen in an AN who continued with their morning personal care work even if a resident resisted verbally, both in the mother tongue and in Swedish, or banged her hands on the table. One resident said in the mother tongue, “Oh, she does not care …” and then fell quiet when the AN continued to wash and undress the resident against her will. An AN could even show disrespect to a resident by taking away food before the resident had finished eating, making the resident upset.

#### Differences between Contexts in the Uncaring Encounters

There were *uncaring encounters* in both contexts (4,8% of notes in the Finnish context, 22% of notes in the Swedish context). Categories that occurred only in the Swedish-speaking context were: not being invited into the conversation (2,2% of notes), providing limited information (2,2% of notes), performing routine work (1,6% of notes), caring against the other’s will (3,3% of notes) and lack of respect for the other (1,6% of notes). Hard-handedness in touch occurred (0,4% of notes) in the Finnish-speaking but not in the Swedish-speaking context.

## Discussion

The results showed the presence of *caring and uncaring encounters* in both the Finnish- and the Swedish-speaking group homes for people with dementia symptoms. Caring encounters were characterized by the existence of conversations between AN and residents, and use of humor, so that residents were adaptable to the ANs’ requests, which were adjusted so that residents with limitations in language abilities could understand. Further, the ANs’ understanding of needs of care was based on every individual resident. Uncaring encounters were characterized by a lack of conversations between AN and the residents or a lack of understanding questions and appeals by residents with limited language abilities. Uncaring encounters also appeared when work was carried out in a routinely manner and when the resident’s will was neglected.

Previous research shows that many older immigrants are cared for in units where no or few ANs speak their mother tongue (Runci et al. 2005; Wu et al. 2010), which complies partly with the conditions of our study. In the Finnish-speaking group home, which was the only one of its kind in the geographic area where the study was conducted, all ANs were bilingual, and in the Swedish-speaking group home, one-third of the ANs were bilingual. The prerequisite for providing high quality care that is based on the person with dementia’s symptoms seen as a unique individual (Sellevold et al. 2013) becomes a challenge. This is because the possibility of using communication to know the resident as a person and which needs the resident has, depends on the language used. It also challenges the opportunity to provide person-centered care in accordance with national guidelines in Sweden. The creation of relationships through communication is a basic requirement for caring encounters (Halldórsdóttir 1996, 2008). Observations in this study showed that the residents’ language and speech were affected due to the disease. This has been described in previous research about the linguistic ability and/or understanding of language for immigrants with dementia symptoms (Tang-Wai and Graham 2008), but to varying degrees depending on the resident’s illness level. Some of the residents were able to hold conversations with the AN, whereas others did not give verbal responses, even when spoken to in the mother tongue. This in turn could affect the outcome of the encounters between AN and residents.

Sellevold (Sellevold et al. 2013) suggested that for high-quality health care, it is important to understand the non-verbal signs from a person with dementia symptoms. Our results show that to facilitate caring encounters, both AN and residents used body language to clarify and supplement the verbal aspect of communication. There were also encounters in which no one spoke to the residents, sat down with the residents at mealtime or invited the residents into the conversation, which led to uncaring encounters. From the AN’s perspective, it is conceivable that the lack of shared language could be one of the reasons for uncaring encounters, a limited attentiveness to the resident’s specific needs, when the resident was not asked about drinks or when information to residents was restricted due to limited language abilities. The older immigrants with dementia symptoms were then at risk of being isolated in a sociolinguistic landscape, as described in previous research (Divita 2014). The shared language, which in our results was a way to create togetherness, was categorized only in the Swedish-speaking context. It might be that the shared language was obvious in the Finnish-speaking context in which everyone had the same mother tongue and therefore it failed to materialize in the results. In the Finnish-speaking group home in which the AN and the residents had the same mother tongue, conversations could be personalized by using humor and Finnish idioms. We cannot know though, if the differences also had to do with the personalities of the nursing staff. Even without the shared language, caregivers could use a playful gesture as shown by Small (Small et al. 2015) in their study of long-term residential care. The result showed that conversations about past life events and daily events at the group home were possible because of the shared language. This, the use of humor and idioms, might have facilitated caring encounters.

Research by Ekman (Ekman 1994) and Söderman and Rosendahl (Söderman and Rosendahl 2016) showed that residents with a lack of communication in foreign languages but who were adequate in their mother tongue could function at a higher level of competence when AN were bilingual. Our results from the observation of the AN instructing a resident while shaving indicate that residents could function at a higher competence level. It might be possible that those residents who showed no reaction when spoken to in their mother tongue had reached such an advanced stage of their disease that verbal communication was no longer possible. Thoughts are clothed in words and symbols through communication (Tang-Wai and Graham 2008), and thus, communication can be a challenge for AN if residents are in an advanced phase of the disease and when they do not have a shared language. Limited communication might be thought to increase ANs´ workload, which has been shown in previous studies on the care of migrants in Sweden (Ekman 1994; Heikkilä et al. 2007; Plejert et al. 2014; Söderman and Rosendahl 2016). Dementia symptoms themselves can hinder the residents’ responses, and although responses can be done through body language they can remain undetected by the observer, or the person being observed might simply have a bad day and not communicate non-verbally. The difference could also be attributed to the individual caregiver who might have known the resident more intimately than another AN. Uncaring encounters in which residents are given limited information and are cared for against their will might be strongly linked to challenges in the care of people with dementia symptoms.

The results in this study confirms previous research that during caring encounters, AN adapted the care for immigrants with dementia symptoms (Jansson 2014; Small et al. 2015). They coaxed, diverted and spoke slowly, so the immigrant with dementia symptoms and language limitations would understand. The AN gave instructions being sensitive to the residents’ reactions and interrupted activity when a resident expressed resistance. The residents were addressed by name, and the AN were considerate about their appearance. This shows that the AN had competence in the care of immigrants with dementia symptoms; they were caring, and there was a connection between the residents and the AN which, according to Halldórsdóttir (Halldórsdóttir 2008, 1996), are the basics of professional caring. Furthermore, the results revealed that during uncaring encounters, there was no or sparse conversation between the residents and AN, work was done by routine and continued against the residents’ will. During the observations, communication was limited, and there was a lack of connection between the residents and AN. It might be that the care was not based on the residents needs because the AN could not interpret their needs. Limitations in language abilities and the lack of a shared language can lead to the risk of needs not being interpreted correctly, as revealed in the study by Plejert (Plejert et al. 2014) and Söderman and Rosendahl (Söderman and Rosendahl 2016). According to Halldórsdóttir (Halldórsdóttir 1996, 2008), encounters characterized by a lack of communication or connection between the residents and AN means that caring becomes uncaring.

A comparison of the number of notes for caring and uncaring encounters showed that uncaring encounters occurred almost only in the Swedish-speaking group home. These occurred in situations in which verbal communication was inadequate, when the AN did not appeal to the resident or when the resident did not understand what the AN said. Uncaring encounters also occurred when the AN did not understand when residents tried to indicate that they wanted a nursing activity to end. It may be a result of residents at the Swedish-speaking group home having a more advanced stage of disease. But it also shows the importance of a shared language and that caring can be a challenge when the shared language is more or less missing. In turn, observations of the Finnish-speaking group home, which had only one note related to an uncaring encounter, proving that caring was facilitated when AN and residents had a shared language. Halldórsdóttir (Halldórsdóttir 1996) considers caring and uncaring not as a dichotomy but a fundamental way of being with another. She describes five ways on the caring and uncaring spectrum; the life-giving mode, life-sustaining mode, life neutral, life-restraining mode and the life-hurting mode.

## Methodological Considerations

Because the purpose of this study was to describe not simply encounters, but caring and uncaring encounters, between the AN and residents, a qualitative deductive approach was suitable for gaining a deeper knowledge than with a quantitative or an inductive approach. The data consisted of field notes from observations, which was well-suited for the purpose. This since observations can focus on verbal communication for different activities or interactions between people. The observations allowed for the capture of behaviors and events, even if the investigator’s presence affected those observed. An alternative way to collect data, using video recording, was considered, but due to financial and ethical issues, it was evaluated that the data based on observations was rich enough to answer the aim of this study. To create a representative survey group that is rich in information (cf. Polit and Beck 2011), the intention was to have two different contexts with many AN and residents to counteract the irregularity of the study group. A difference was that in the Finnish-speaking context, everyone was observed, although some appeared more often in observations, which was not the intention. In the Swedish-speaking context, the observations could be insufficient because the focus was on selected residents who were not Swedish-speaking, and therefore a part of the interaction could have been lost. There was also a difference between the notes from the two contexts: Those from the Finnish-speaking context were more precise than the others, and might be more positively related to the observer’s Finnish origin. However, the observer was aware of this possibility and thus safeguarded against such a pitfall by keeping a diary before and after the observations. A qualitative, deductive content analysis enabled a variation in the descriptions of encounters and a reduction in preconceptions, influences and interpretations, and an analysis framework validated by discussions between the authors (MS, CS) was used. Credibility was reached by discussions between the authors (MS, CS) throughout the analysis process. During the observations, the researcher was focused on writing down as much as possible of what was observed of the ongoing communication and interaction and did not specifically look for caring aspects so the reflection over the caring at group homes began when the material was read and analyzed with the help of a theory. Thoughts that arose were that the researcher also had witnessed uncaring, which could be perceived as complex even for a registered nurse working at the unit.

## Conclusion

The person-centered approach is based on communication and creation of relationships (Sjögren et al. 2013), which is also the basis for caring encounters. The theory of caring and uncaring encounters (Halldórsdóttir 1996, 2008) was chosen to describe the daily encounters between AN and immigrants with dementia symptoms. The theory was useful in the context chosen for this study and results showed that encounters could be both caring and uncaring in group homes for persons with dementia symptoms. Encounters could also be carried out using a person-centered approach by seeing the person with dementia symptoms and their needs and base the care on those needs. Prerequisites for this emerged; for example, caring encounters could be facilitated by having a shared language and thus being able to communicate with each other and form relationships, even for those with severe dementia symptoms. When there was not a shared language, relationships could be created between the caregiver and the immigrant with dementia symptoms when an AN had an increased responsibility and commitment to a resident and thus learned to interpret the resident’s needs and desires.

Through increased knowledge of the theory of caring and uncaring encounters applied in a group home for people with dementia symptoms, AN might be given support to be able to reflect on the care they give. This process of reflection could be guided by RN. With increased knowledge and understanding, AN might then have more purposeful encounters and be better prepared for the encounters they might face during their daily work. Not considering an encounter or not taking a position in an encounter can lead to an uncaring encounter. However, through reflection, AN have the opportunity to take a stand with the intention of creating a caring encounter. Although the observations in the present study were conducted in group homes for people with dementia symptoms, knowledge of caring and uncaring encounters provides support for AN during encounters with other people who are in a vulnerable situation.
